# Supplementary material for: MICROGLIA AGING IN THE HIPPOCAMPUS ADVANCES THROUGH INTERMEDIATE STATES THAT DRIVE ACTIVATION AND COGNITIVE DECLINE
Source: bioRxiv. 2024 Dec 19:2024.04.09.588665. Originally published 2024 Apr 9. Preprint. [Version 2] doi: 10.1101/2024.04.09.588665 (PMC11030314; doi:10.1101/2024.04.09.588665)
Supplement: Supplement 1 — Fig. S1 | Hippocampal microglia exhibit age-related heterogeneity during aging. A, UMAP plot of all Cd11b+ cells. Several adjacent clusters of microglia were identified, as well as a cluster of proliferating microglia. Smaller populations of peripheral immune cell types – macrophages and neutrophils – were identified. Clusters of astrocytes and vascular cells were also found. Overall, greater than 82% of cells were microglia. (n = 1 pool of 5 animals for each age) B, Dot plot showing expression (average expression and percent of cells expressing) of top two markers for each cluster. C, UMAP plots with expression levels of microglia markers superimposed onto cells. Notice that peripheral immune cells express microglia markers; however, they are distinguished from microglia based on marker expression from (b). D-G, Volcano plots of differential gene expression for the clusters identified in Figure 1A compared to every other cluster for Homeostatic (D), Transition (E), Activation (F), and Interferon (G) microglia clusters. H, Cluster composition of non-proliferating microglia by age. I, Standardized variation of non-proliferating microglia for each age. Fig. S2 | Spatiotemporal kinetics of microglial inflammatory activation in the aging hippocampus. A, Diagram depicting ages utilized for immunohistochemical analysis. B, Diagram of the hippocampus labeled with the regions analyzed. C, Illustration of subregions analyzed by immunohistochemistry. D, Representative images and quantification of IBA1 (cyan)/CD68 (red)-positive microglia across hippocampal subregions in 3- and 24-month old mice. Scale bars are 10μM. (n=5 per group; T-test with Holm-Sidak correction; *P<0.05, **P<0.01) E, Heatmap of the quantification of activated microglia across ages and subregions. F, Representative wide field images of IBA1 (cyan)/CD68 (red) in the dentate gyrus in 3- and 24-month old mice. Scale bars are 100μM. G, Representative wide field images of Iba1 (cyan)/ NFKB p65 (yellow) in the d [file media-1.pdf]

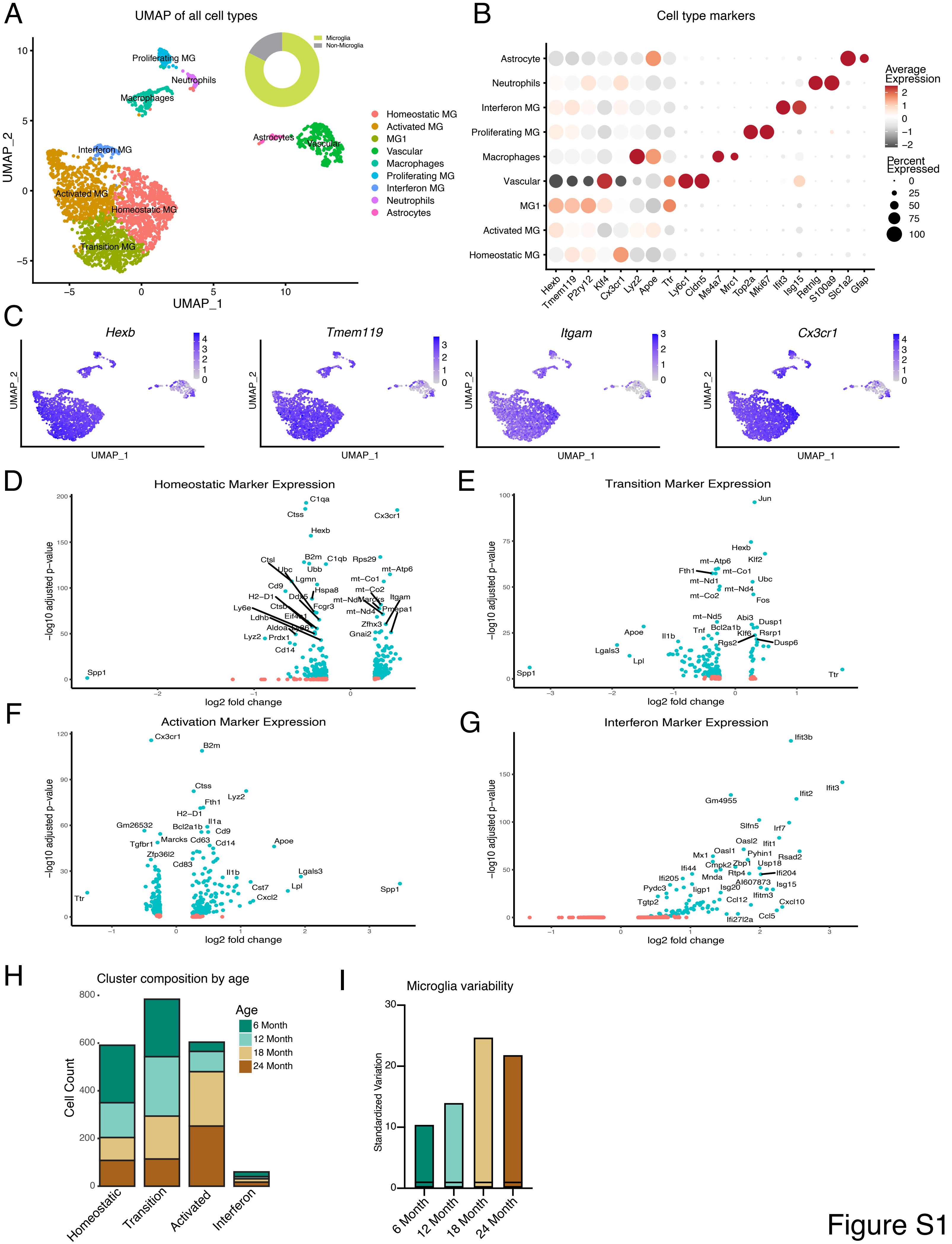

A
UMAP of all cell types
Microglia
Non-Microglia
Proliferating MG
Neutrophils
Macrophages
Interferon MG
Astrocytes
Vascular
Activated MG
Homeostatic MG
Transition MG
UMAP_1
UMAP_2
Homeostatic MG
Activated MG
MG1
Vascular
Macrophages
Proliferating MG
Interferon MG
Neutrophils
Astrocytes
B
Cell type markers
Astrocyte
Neutrophils
Interferon MG
Proliferating MG
Macrophages
Vascular
MG1
Activated MG
Homeostatic MG
Hexb
Tmem119
P2ry12
Klf4
Cx3cr1
Lyz2
Apoe
Ttr
Ly6c1
Cldn5
Ms4a7
Mrc1
Top2a
Mki67
Ifit3
Isg15
Retnlg
S100a9
Slc1a2
Gfap
Average Expression
Percent Expressed
C
Hexb
Tmem119
Itgam
Cx3cr1
D
Homeostatic Marker Expression
-log10 adjusted p-value
log2 fold change
E
Transition Marker Expression
F
Activation Marker Expression
G
Interferon Marker Expression
H
Cluster composition by age
Cell Count
Age
6 Month
12 Month
18 Month
24 Month
Homeostatic
Transition
Activated
Interferon
I
Microglia variability
Standardized Variation
6 Month
12 Month
18 Month
24 Month

Figure S1

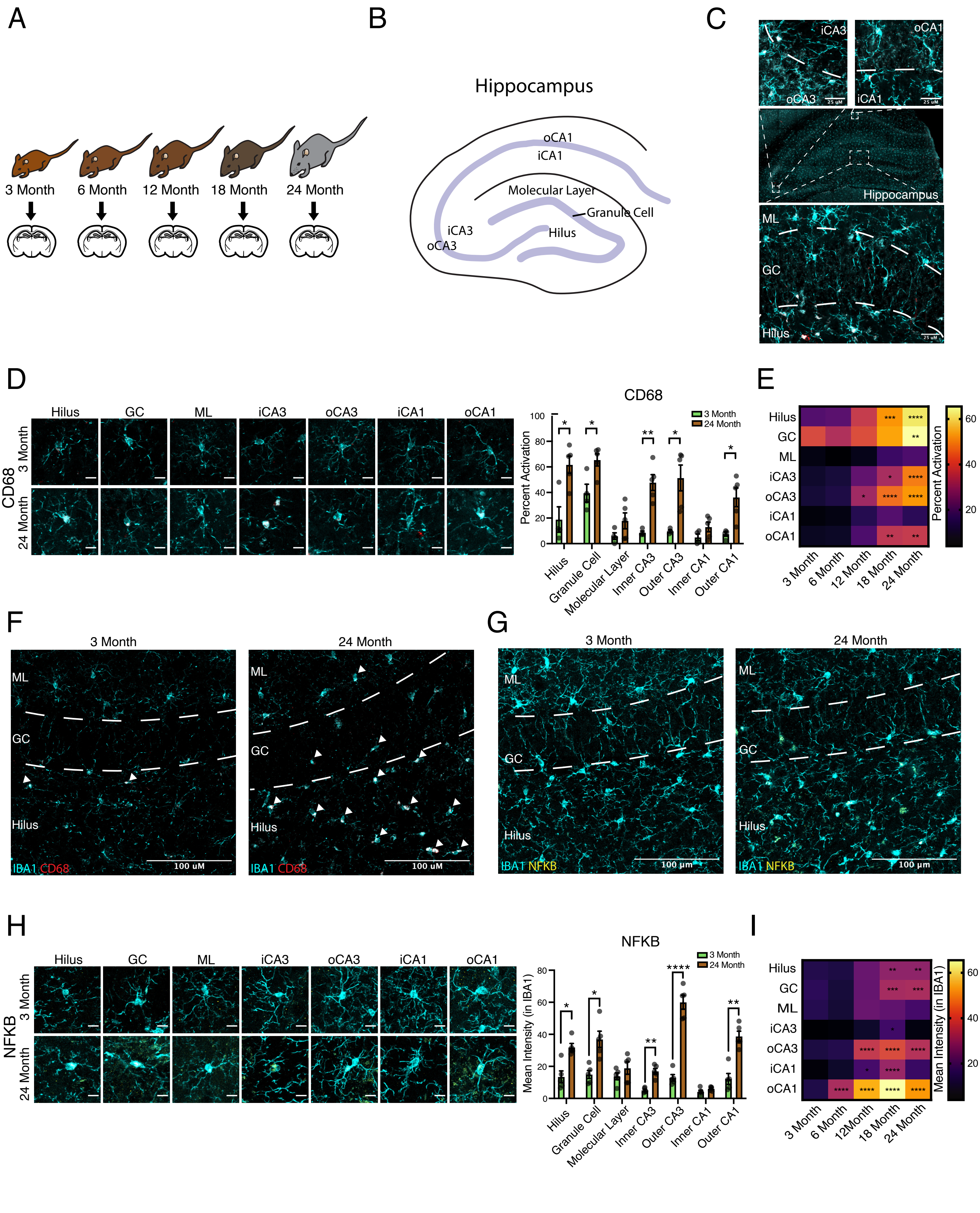

A
3 Month
6 Month
12 Month
18 Month
24 Month
B
Hippocampus
oCA1
iCA1
Molecular Layer
Granule Cell
iCA3
oCA3
Hilus
C
iCA3
oCA3
oCA1
iCA1
Hippocampus
ML
GC
Hilus
D
Hilus
GC
ML
iCA3
oCA3
iCA1
oCA1
CD68
3 Month
24 Month
CD68
Percent Activation
3 Month
24 Month
Granule Cell
Molecular Layer
Inner CA3
Outer CA3
Inner CA1
Outer CA1
E
Percent Activation
F
3 Month
24 Month
IBA1 CD68
100 uM
G
IBA1 NFKB
100 µm
H
NFKB
Mean Intensity (in IBA1)
I
12Month

Figure S2

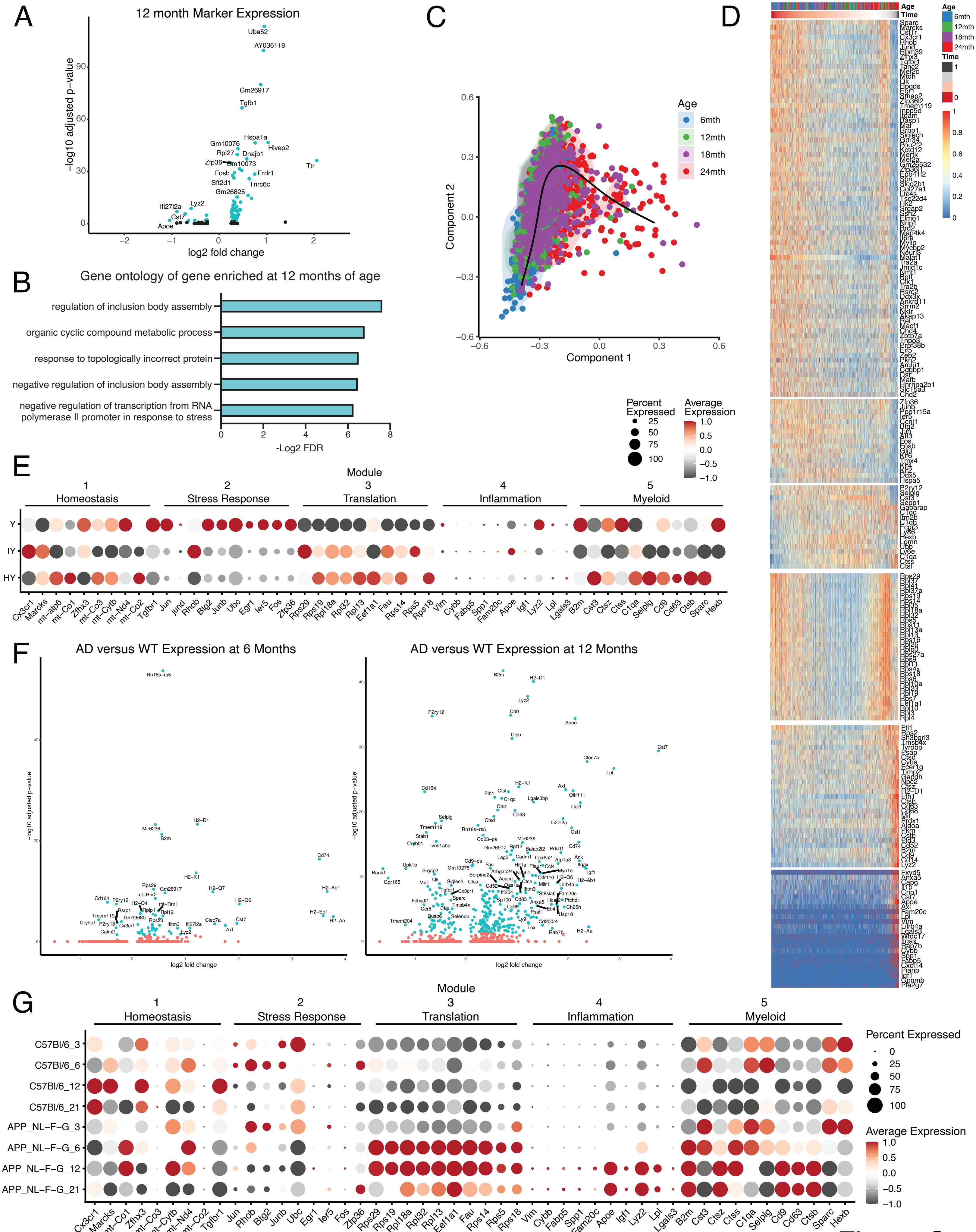
A
12 month Marker Expression
−log10 adjusted p−value
log2 fold change
B
Gene ontology of gene enriched at 12 months of age
regulation of inclusion body assembly
organic cyclic compound metabolic process
response to topologically incorrect protein
negative regulation of inclusion body assembly
negative regulation of transcription from RNA polymerase II promoter in response to stress
-Log2 FDR
C
Component 1
Component 2
Age
6mth
12mth
18mth
24mth
D
Age
Time
E
Module
1 Homeostasis
2 Stress Response
3 Translation
4 Inflammation
5 Myeloid
Percent Expressed
Average Expression
F
AD versus WT Expression at 6 Months
AD versus WT Expression at 12 Months
G
Module
1 Homeostasis
2 Stress Response
3 Translation
4 Inflammation
5 Myeloid
C57Bl/6_3
C57Bl/6_6
C57Bl/6_12
C57Bl/6_21
APP_NL−F−G_3
APP_NL−F−G_6
APP_NL−F−G_12
APP_NL−F−G_21
Percent Expressed
Average Expression

Figure S3

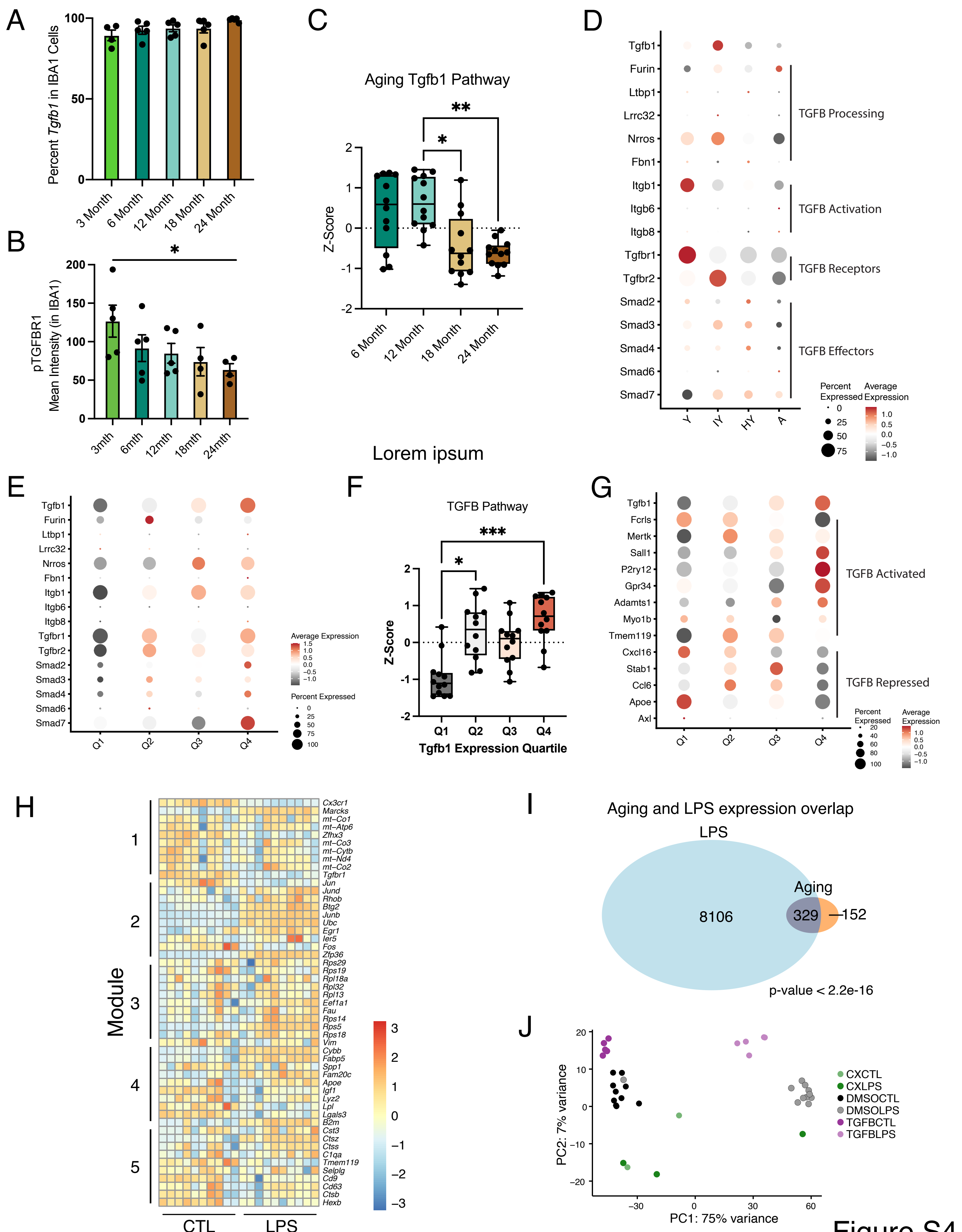
A
Percent Tgfb1 in IBA1 Cells
3 Month
6 Month
12 Month
18 Month
24 Month
B
pTGFBR1 Mean Intensity (in IBA1)
3mth
6mth
12mth
18mth
24mth
C
Aging Tgfb1 Pathway
Z-Score
6 Month
12 Month
18 Month
24 Month
D
Tgfb1
Furin
Ltbp1
Lrrc32
Nrros
Fbn1
Itgb1
Itgb6
Itgb8
Tgfbr1
Tgfbr2
Smad2
Smad3
Smad4
Smad6
Smad7
TGFB Processing
TGFB Activation
TGFB Receptors
TGFB Effectors
Y
IY
HY
A
Percent Expressed
Average Expression
Lorem ipsum
E
Average Expression
Percent Expressed
Q1
Q2
Q3
Q4
F
TGFB Pathway
Z-Score
Tgfb1 Expression Quartile
G
Tgfb1
Fcrls
Mertk
Sall1
P2ry12
Gpr34
Adamts1
Myo1b
Tmem119
Cxcl16
Stab1
Ccl6
Apoe
Axl
TGFB Activated
TGFB Repressed
H
Module
Cx3cr1
Marcks
mt-Co1
mt-Atp6
Zfhx3
mt-Co3
mt-Cytb
mt-Nd4
mt-Co2
Tgfbr1
Jun
Jund
Rhob
Btg2
Junb
Ubc
Egr1
Ier5
Fos
Zfp36
Rps29
Rps19
Rpl18a
Rpl32
Rpl13
Eef1a1
Fau
Rps14
Rps5
Rps18
Vim
Cybb
Fabp5
Spp1
Fam20c
Apoe
Igf1
Lyz2
Lpl
Lgals3
B2m
Cst3
Ctsz
Ctss
C1qa
Tmem119
Selplg
Cd9
Cd63
Ctsb
Hexb
CTL
LPS
I
Aging and LPS expression overlap
LPS
Aging
8106
329
152
p-value < 2.2e-16
J
PC2: 7% variance
PC1: 75% variance
CXCTL
CXLPS
DMSOCTL
DMSOLPS
TGFBCTL
TGFBLPS

Figure S4

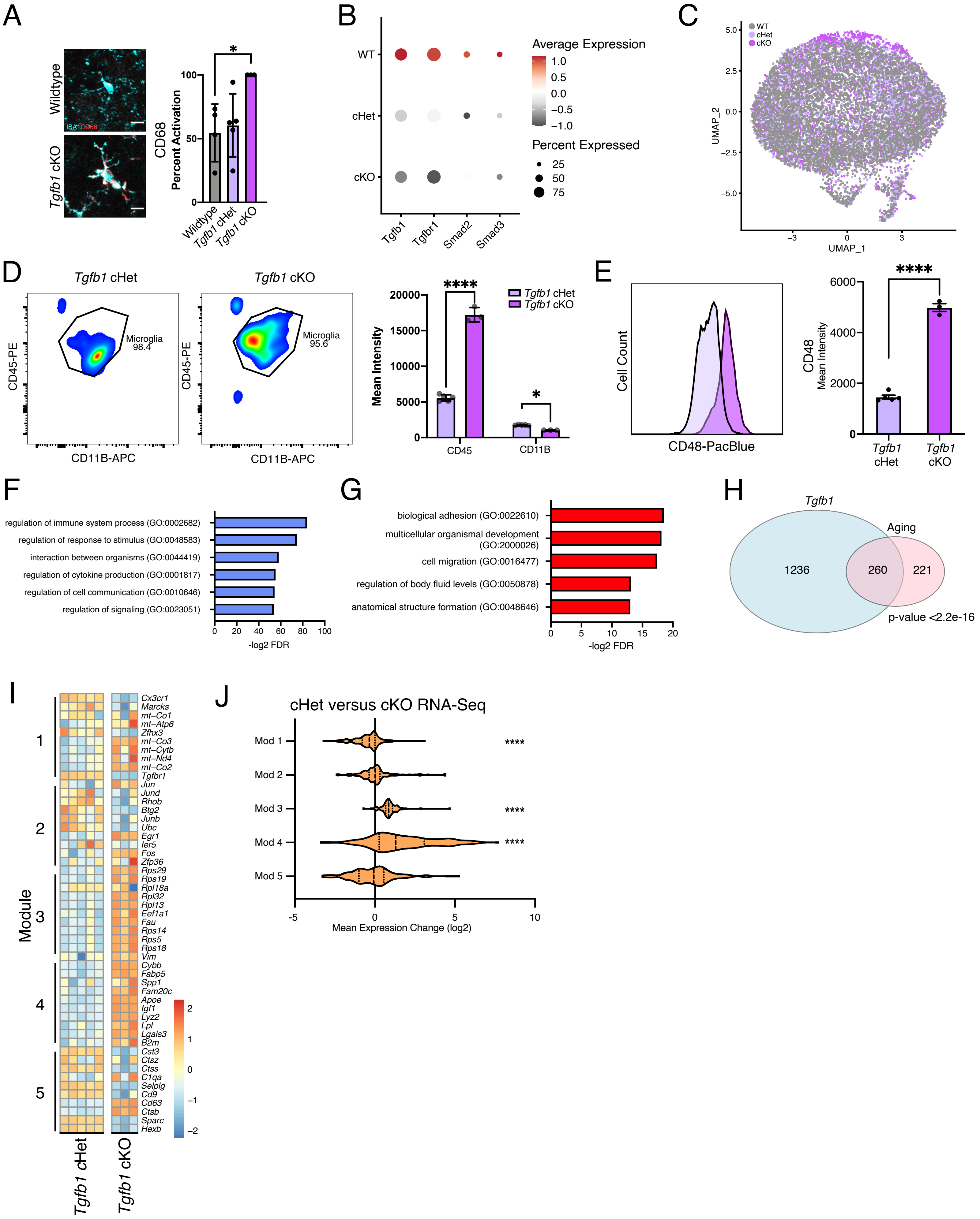
A
Wildtype
Tgfb1 cKO
IBA1 CD68
CD68
Percent Activation
100
50
0
Wildtype
Tgfb1 cHet
Tgfb1 cKO
*
B
WT
cHet
cKO
Tgfb1
Tgfbr1
Smad2
Smad3
Average Expression
1.0
0.5
0.0
−0.5
−1.0
Percent Expressed
25
50
75
C
WT
cHet
cKO
UMAP_2
UMAP_1
D
Tgfb1 cHet
Tgfb1 cKO
Microglia
98.4
95.6
CD45-PE
CD11B-APC
Mean Intensity
Tgfb1 cHet
Tgfb1 cKO
CD45
CD11B
****
*
E
Cell Count
CD48-PacBlue
CD48
Mean Intensity
Tgfb1 cHet
Tgfb1 cKO
****
F
regulation of immune system process (GO:0002682)
regulation of response to stimulus (GO:0048583)
interaction between organisms (GO:0044419)
regulation of cytokine production (GO:0001817)
regulation of cell communication (GO:0010646)
regulation of signaling (GO:0023051)
-log2 FDR
G
biological adhesion (GO:0022610)
multicellular organismal development (GO:2000026)
cell migration (GO:0016477)
regulation of body fluid levels (GO:0050878)
anatomical structure formation (GO:0048646)
-log2 FDR
H
Tgfb1
Aging
1236
260
221
p-value <2.2e-16
I
Module
1
2
3
4
5
Cx3cr1
Marcks
mt-Co1
mt-Atp6
Zfhx3
mt-Co3
mt-Cytb
mt-Nd4
mt-Co2
Tgfbr1
Jun
Jund
Rhob
Btg2
Junb
Ubc
Egr1
Ier5
Fos
Zfp36
Rps29
Rps19
Rpl18a
Rpl32
Rpl13
Eef1a1
Fau
Rps14
Rps5
Rps18
Vim
Cybb
Fabp5
Spp1
Fam20c
Apoe
Igf1
Lyz2
Lpl
Lgals3
B2m
Cst3
Ctsz
Ctss
C1qa
Selplg
Cd9
Cd63
Ctsb
Sparc
Hexb
Tgfb1 cHet
Tgfb1 cKO
J
cHet versus cKO RNA-Seq
Mod 1
Mod 2
Mod 3
Mod 4
Mod 5
****
Mean Expression Change (log2)

Figure S5

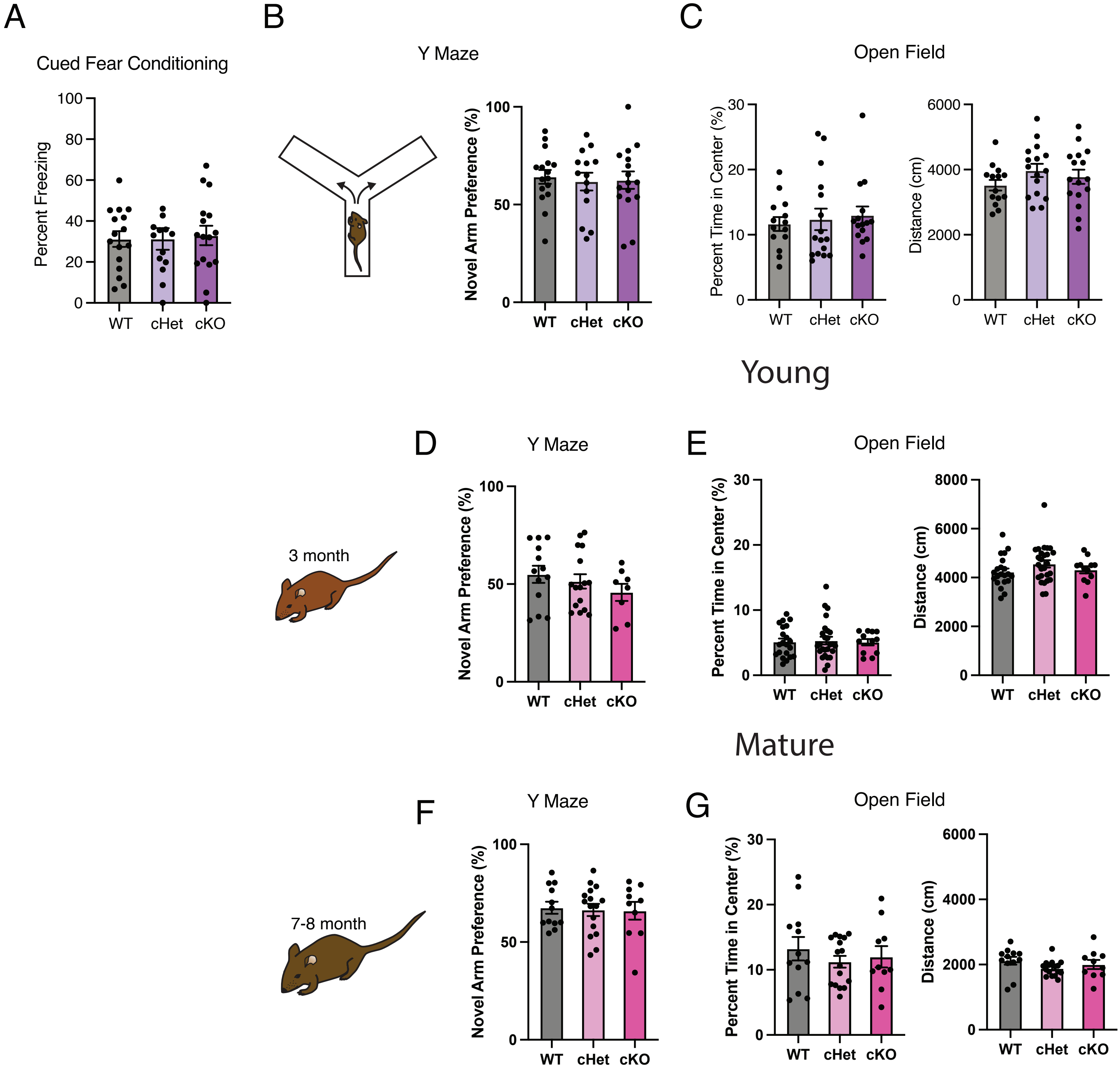
A
Cued Fear Conditioning
Percent Freezing
100
80
60
40
20
0
WT
cHet
cKO
B
Y Maze
Novel Arm Preference (%)
100
50
0
WT
cHet
cKO
C
Open Field
Percent Time in Center (%)
30
20
10
0
WT
cHet
cKO
Distance (cm)
6000
4000
2000
0
WT
cHet
cKO
Young
3 month
D
Y Maze
Novel Arm Preference (%)
100
50
0
WT
cHet
cKO
E
Open Field
Percent Time in Center (%)
30
20
10
0
WT
cHet
cKO
Distance (cm)
8000
6000
4000
2000
0
WT
cHet
cKO
Mature
7-8 month
F
Y Maze
Novel Arm Preference (%)
100
50
0
WT
cHet
cKO
G
Open Field
Percent Time in Center (%)
30
20
10
0
WT
cHet
cKO
Distance (cm)
6000
4000
2000
0
WT
cHet
cKO

Figure S6
