# Supplementary material for: MICROGLIA AGING IN THE HIPPOCAMPUS ADVANCES THROUGH INTERMEDIATE STATES THAT DRIVE ACTIVATION AND COGNITIVE DECLINE
Source: bioRxiv. 2024 Dec 19:2024.04.09.588665. Originally published 2024 Apr 9. Preprint. [Version 2] doi: 10.1101/2024.04.09.588665 (PMC11030314; doi:10.1101/2024.04.09.588665)
Supplement: Supplement 2 [file NIHPP2024.04.09.588665v2-supplement-2.pdf]

bioRxiv preprint doi: https://doi.org/10.1101/2024.04.09.588665; this version posted January 3, 2025. The copyright holder for this preprint (which was not certified by peer review) is the author/funder, who has granted bioRxiv a license to display the preprint in perpetuity. It is made available under aCC-BY 4.0 International license.

Table S1 | Gene markers for each age group and differential expression between the 6- and 12-, 18-, or 24-month-old microglia in single-cell RNA-Seq of aging hippocampal microglia. The tables contain genes selectively increased or decreased in each age group when compared to every other age group combined and genes differentially expressed between 6- and 12-, 18-, or 24-month microglia. The tables contain the average log fold change, the percentage of cells in each group with expression, and the adjusted p-value.

bioRxiv preprint doi: https://doi.org/10.1101/2024.04.09.588665; this version posted January 3, 2025. The copyright holder for this preprint (which was not certified by peer review) is the author/funder, who has granted bioRxiv a license to display the preprint in perpetuity. It is made available under aCC-BY 4.0 International license.

Table S2 | Pseudotime trajectories of aging microglia. The pseudotime analysis contains the spatial autocorrelation analysis. Moran's I was used to detect focal expression of genes that were consequently constructed into co-regulated modules using Louvain community analysis. The table contains the adjusted p-value (q-value) and module information.

bioRxiv preprint doi: https://doi.org/10.1101/2024.04.09.588665; this version posted January 3, 2025. The copyright holder for this preprint (which was not certified by peer review) is the author/funder, who has granted bioRxiv a license to display the preprint in perpetuity. It is made available under aCC-BY 4.0 International license.

Table S3 | RNA-Seq analysis of primary microglia treated with LPS and TGFB1 or CX-5461. The tables contain the RNA-Seq differential expression analysis of control and LPS activated primary microglia, DMSO and CX-5461 treated primary microglia activated by LPS, and DMSO and TGFB1 treated primary microglia activated by LPS, respectively. The tables contain expression values of genes for the samples that were compared along with mean expression values for all samples combined, log fold change between sample groups, and adjusted p-values. in the RNA-Seq differential expression analysis of control and LPS activated primary microglia, DMSO and CX-5461 treated primary microglia activated by LPS, and DMSO and TGFB1 treated primary microglia activated by LPS, respectively.

bioRxiv preprint doi: https://doi.org/10.1101/2024.04.09.588665; this version posted January 3, 2025. The copyright holder for this preprint (which was not certified by peer review) is the author/funder, who has granted bioRxiv a license to display the preprint in perpetuity. It is made available under aCC-BY 4.0 International license.

Table S4 | Differential expression analysis of control and *Tgfb1* cKO hippocampal microglia. The table contains expression values of genes for the samples that were compared along with mean expression values for all samples combined, log fold change between sample groups, and adjusted p-values.
