# Supplementary material for: MICROGLIA AGING IN THE HIPPOCAMPUS ADVANCES THROUGH INTERMEDIATE STATES THAT DRIVE INFLAMMATORY ACTIVATION AND COGNITIVE DECLINE
Source: bioRxiv. 2024 Apr 9:2024.04.09.588665. Preprint. [Version 1] doi: 10.1101/2024.04.09.588665 (PMC11030314; doi:10.1101/2024.04.09.588665)
Supplement: Supplementary file 1 [file NIHPP2024.04.09.588665v1-supplement-1.pdf]

bioRxiv preprint doi: https://doi.org/10.1101/2024.04.09.588665; this version posted April 9, 2024. The copyright holder for this preprint (which was not certified by peer review) is the author/funder, who has granted bioRxiv a license to display the preprint in perpetuity. It is made available under aCC-BY 4.0 International license.

SUPPLEMENTAL MATERIAL

Fig. S1 | Hippocampal microglia exhibit age-related heterogeneity during aging. A, UMAP plot of all Cd11b+ cells. Several adjacent clusters of microglia were identified, as well as a cluster of proliferating microglia. Smaller populations of peripheral immune cell types – macrophages and neutrophils – were identified. Clusters of astrocytes and vascular cells were also found. Overall, greater than 82% of cells were microglia. (n = 1 pool of 5 animals for each age) **B,** Dot plot showing expression (average expression and percent of cells expressing) of top two markers for each cluster. **C,** UMAP plots with expression levels of microglia markers superimposed onto cells. Notice that peripheral immune cells express microglia markers; however, they are distinguished from microglia based on marker expression from (b). **D-G,** Volcano plots of differential gene expression for the clusters identified in Figure 1A compared to every other cluster for Homeostatic (**D**), Transition (**E**), Activation (**F**), and Interferon (**G**) microglia clusters. **H,** Cluster composition of non-proliferating microglia by age. **I,** Standardized variation of non-proliferating microglia for each age. **J,** Representative images of NFκB (p65) (yellow) and IBA1 (cyan) staining in the hippocampus of 3- and 24-month-old mice and quantification of staining in 3-, 6-, 12-, 18-, and 24-month-old mice. (n=5 mice per group; one-way ANOVA with Dunnett's post hoc test; *P<0.05, ***P<0.001)**. K,** Representative images of CD68 (red) and IBA1 (cyan) staining in the hippocampus of 3- and 24-month-old mice and quantification of staining in 3-, 6-, 12-, 18-, and 24-month-old mice. (n=4-5 mice per group; one-way ANOVA with Dunnett's post hoc test; *P<0.05, **P<0.01)**.** Data are shown as mean±s.e.m.

bioRxiv preprint doi: https://doi.org/10.1101/2024.04.09.588665; this version posted April 9, 2024. The copyright holder for this preprint (which was not certified by peer review) is the author/funder, who has granted bioRxiv a license to display the preprint in perpetuity. It is made available under aCC-BY 4.0 International license.

Figure S2 | Hippocampal microglia aging advances through intermediate states that respond to systemic interventions or disease states. **A**, Volcano plot of differential gene expression of 12-month microglia versus all other ages with significant genes in teal. **B**, Gene ontology analysis of biological processes enriched in those genes with increased expression at 12-months of age. **C**, Representative images and quantification of CD68 (red) and IBA1 (cyan) staining in the hippocampus of isochronic young (IY) and heterochronic young (HY) along with a diagram of the comparisons. (n=5 mice per group; unpaired Student's T-test; ***P<0.001)**.** Data are shown as mean±s.e.m. **D,** Dotplot of pseudotime modules in young (Y), isochronic young (IY), and heterochronic young (HY) parabiont microglia. Data is from Palovics *et al*[61]. Percent of cells expressing the gene and average normalized expression are represented. **E,** Volcano plots of differential gene expression of *App*$^{NL\text{-}G\text{-}F}$ genotype (AD) over wildtype (WT) microglia at 6- (left) and 12-months of age. Significant genes are in teal. Data is from Frigerio[62]. **F,** Dotplot of pseudotime modules across ages (3-, 6-, 12-, and 21-months old) and genotypes (*App*$^{NL\text{-}G\text{-}F}$ genotype (AD) over C57Bl/6 (WT)). Data is from Frigerio[17]. Percent of cells expressing the gene and average normalized expression are represented.

bioRxiv preprint doi: https://doi.org/10.1101/2024.04.09.588665; this version posted April 9, 2024. The copyright holder for this preprint (which was not certified by peer review) is the author/funder, who has granted bioRxiv a license to display the preprint in perpetuity. It is made available under aCC-BY 4.0 International license.

Fig. S3 | Intermediate states of microglia aging act as checkpoints on inflammatory progression. A, Quantification of percentage of *Tgfb1* RNAscope signal within IBA1 cells. **B**, Quantification of IHC of pTGFBR1 signal in IBA1 cells across ages. (n=4-5 mice per group; one-way ANOVA; *P<0.05) **C,** Dotplot of the expression values of TGFB1 signaling components from scRNA-Seq of aging hippocampal microglia (6-, 12-, 18-, and 24-month-old). Percent of cells expressing the gene and average normalized expression are represented. **D**, Dotplot of the expression values of TGFB1 signaling components in young (Y), aged (A), isochronic young (IY), and heterochronic young (HY) parabiont microglia. Data is from Palovics *et al*[61]. Percent of cells expressing the gene and average normalized expression are represented. UMAP plot of *Tgfb1* scRNA-Seq colored by genotype. **E**, Heatmap of gene expression changes in the top 10 genes in each aging module induced by LPS. **F**, Overlap between microglia gene expression changes induced by LPS and aging. (χ^2<2.2e-16). **G**, PCA plot of pharmacological manipulations with or without LPS treatment.

bioRxiv preprint doi: https://doi.org/10.1101/2024.04.09.588665; this version posted April 9, 2024. The copyright holder for this preprint (which was not certified by peer review) is the author/funder, who has granted bioRxiv a license to display the preprint in perpetuity. It is made available under aCC-BY 4.0 International license.

Fig. S4 | Targeting age-related changes in microglia-derived TGFB1 promotes microglia advancement along inflammatory trajectories in the hippocampus and impairs cognition. **A**, Representative images and quantification of IBA1 (cyan)/CD68 (red)-positive microglia in wildtype, *Tgfb1* cHet, and *Tgfb1* cKO hippocampi. (n=3-5; one way ANOVA with Tukey post hoc test; *P<0.05) **B,** Dotplot of the expression values of TGFB1 signaling components from scRNA-Seq of *Tgfb1* WT, cHet, and cKO microglia. (n = 2 pools of 3 animals per genotype) **C,** UMAP plot of *Tgfb1* scRNA-Seq colored by genotype. **D,** FACS plots of gating strategy for microglia isolation for RNA-Seq in cHet and cKO hippocampi. Quantification of the flow cytometry analysis of CD11b and CD45. (n=3-5; mixed effects analysis; *P<0.05, ****P<0.0001). **E**, Example histogram and quantification of CD48 in control and *Tgfb1* cKO FACS-sorted microglia. (n=3-5 per group; T-test; ****P<0.0001). **F,** Top gene ontology terms associated with genes significantly increased in microglia bulk RNA-Seq in *Tgfb1* cKO samples (n=3-5 samples per genotype). **G**, Top gene ontology terms associated with genes significantly decreased in microglia bulk RNA-Seq in *Tgfb1* cKO samples. **H**, Overlap between concordant microglia gene expression changes induced by *Tgfb1* knockout and aging. (χ^2<2.2e-16). **I**, Heatmap of gene expression of the top 10 genes in each aging module in control and *Tgfb1* cKO microglia. **J**, Average gene expression changes for each aging module represented as log2 fold change of *Tgfb1* cKO over control. (one sample T-test with the expected value of 0 (no change); ****P<0.0001). **K**, Freezing during the cued fear conditioning phase of the fear conditioning test. The percent freezing was calculated for the last 30 seconds of the test following the conditioned stimulus. (n=13-16 per genotype) **L**, Diagram and results of Y-maze test. Results are presented as preference for the novel arm versus the trained armed (time in novel arm/ (time in novel arm + trained arm) x 100). (n=14-16 per genotype) **M**, Percent time in the center of the open field and distance traveled during the open field test. (n=14-16 per genotype) Data are shown as mean±s.e.m.

bioRxiv preprint doi: https://doi.org/10.1101/2024.04.09.588665; this version posted April 9, 2024. The copyright holder for this preprint (which was not certified by peer review) is the author/funder, who has granted bioRxiv a license to display the preprint in perpetuity. It is made available under aCC-BY 4.0 International license.

Table S1 | Gene markers for each age group and differential expression between the 6- and 12-, 18-, or 24-month-old microglia in single-cell RNA-Seq of aging hippocampal microglia. The tables contain genes selectively increased or decreased in each age group when compared to every other age group combined and genes differentially expressed between 6- and 12-, 18-, or 24-month microglia. The tables contain the average log fold change, the percentage of cells in each group with expression, and the adjusted p-value.

bioRxiv preprint doi: https://doi.org/10.1101/2024.04.09.588665; this version posted April 9, 2024. The copyright holder for this preprint (which was not certified by peer review) is the author/funder, who has granted bioRxiv a license to display the preprint in perpetuity. It is made available under aCC-BY 4.0 International license.

Table S2 | Pseudotime trajectories of aging microglia. The pseudotime analysis contains the spatial autocorrelation analysis. Moran's I was used to detect focal expression of genes that were consequently constructed into co-regulated modules using Louvain community analysis. The table contains the adjusted p-value (q-value) and module information.

bioRxiv preprint doi: https://doi.org/10.1101/2024.04.09.588665; this version posted April 9, 2024. The copyright holder for this preprint (which was not certified by peer review) is the author/funder, who has granted bioRxiv a license to display the preprint in perpetuity. It is made available under aCC-BY 4.0 International license.

Table S3 | RNA-Seq analysis of primary microglia treated with LPS and TGFB1 or CX-5461. The tables contain the RNA-Seq differential expression analysis of control and LPS activated primary microglia, DMSO and CX-5461 treated primary microglia activated by LPS, and DMSO and TGFB1 treated primary microglia activated by LPS, respectively. The tables contain expression values of genes for the samples that were compared along with mean expression values for all samples combined, log fold change between sample groups, and adjusted p-values. in the RNA-Seq differential expression analysis of control and LPS activated primary microglia, DMSO and CX-5461 treated primary microglia activated by LPS, and DMSO and TGFB1 treated primary microglia activated by LPS, respectively.

bioRxiv preprint doi: https://doi.org/10.1101/2024.04.09.588665; this version posted April 9, 2024. The copyright holder for this preprint (which was not certified by peer review) is the author/funder, who has granted bioRxiv a license to display the preprint in perpetuity. It is made available under aCC-BY 4.0 International license.

Table S4 | Differential expression analysis of control and *Tgfb1* cKO hippocampal microglia. The table contains expression values of genes for the samples that were compared along with mean expression values for all samples combined, log fold change between sample groups, and adjusted p-values.
